# Microbial Upgrading of Acetate into Value-Added Products—Examining Microbial Diversity, Bioenergetic Constraints and Metabolic Engineering Approaches

**DOI:** 10.3390/ijms21228777

**Published:** 2020-11-20

**Authors:** Regina Kutscha, Stefan Pflügl

**Affiliations:** Institute for Chemical, Environmental and Bioscience Engineering, Research Area Biochemical Engineering, Technische Universität Wien, Gumpendorfer Straße 1a, 1060 Vienna, Austria; regina.kutscha@tuwien.ac.at

**Keywords:** acetate, *Escherichia coli*, acetate metabolism, metabolic engineering, process engineering, acetate tolerance, acetate-derived chemicals, bioenergetic constraints

## Abstract

Ecological concerns have recently led to the increasing trend to upgrade carbon contained in waste streams into valuable chemicals. One of these components is acetate. Its microbial upgrading is possible in various species, with *Escherichia coli* being the best-studied. Several chemicals derived from acetate have already been successfully produced in *E. coli* on a laboratory scale, including acetone, itaconic acid, mevalonate, and tyrosine. As acetate is a carbon source with a low energy content compared to glucose or glycerol, energy- and redox-balancing plays an important role in acetate-based growth and production. In addition to the energetic challenges, acetate has an inhibitory effect on microorganisms, reducing growth rates, and limiting product concentrations. Moreover, extensive metabolic engineering is necessary to obtain a broad range of acetate-based products. In this review, we illustrate some of the necessary energetic considerations to establish robust production processes by presenting calculations of maximum theoretical product and carbon yields. Moreover, different strategies to deal with energetic and metabolic challenges are presented. Finally, we summarize ways to alleviate acetate toxicity and give an overview of process engineering measures that enable sustainable acetate-based production of value-added chemicals.

## 1. Introduction

When producing value-added chemicals or recombinant proteins in *Escherichia coli*, acetate formation can pose challenges. It has long been established that *E. coli* produces acetate together with other fermentation products like lactate and ethanol under anaerobic conditions [1]. However, acetate can also accumulate and cause growth inhibition when cells are grown aerobically with excess glucose (acetate overflow metabolism) [2]. Additionally, it was found that acetate accumulation reduces the yield of recombinantly produced proteins [3,4,5].

The amount of accumulated acetate may vary between different *E. coli* strains. On the one hand, *E. coli W* exhibits only low amounts of accumulated acetate [6,7]. On the other hand, the acetate titers after batch cultivation of the *E. coli* strains JB101, JM105, and B on 20 g/L glucose and a dissolved oxygen level maintained above 20% were reported to be 0.88 g/L, 1.20 g/L, and 1.75 g/L, respectively [8].

To avoid the negative effects of acetate accumulation, extensive research on the cause of overflow metabolism has been done. For example, studies have identified the influence of acetyl-CoA synthetase, the cellular redox ratio, and the proteome efficiency of fermentative pathways [9,10,11,12]. In addition to research on overflow metabolism, the mechanisms of growth inhibition caused by acetate were also thoroughly investigated [13,14,15].

The implementation of different strategies helped to circumvent the challenges posed by acetate. Since *E. coli* BL21(DE3) exhibits a reduced acetate accumulation in glucose batches, recombinant protein production has shifted towards this host strain [16]. Moreover, an alkaline pH shift, the addition of selected amino acids, and the elimination of genes involved in acetate formation were shown to reduce acetate accumulation [17,18,19,20,21].

Although often avoided as a substrate for *E. coli* processes, acetate is a part of many low-cost feedstocks such as lignocellulosic hydrolysates [22,23,24]. For sustainable biotechnological production of chemicals, cheap substrates are required to achieve ecological and economic advantages over fossil fuel-based production. Accordingly, acetate conversion is a vital component for a holistic utilization of all carbon sources in potential feedstocks. Appropriate reviews thereon have already been published [25,26].

In other cases, acetate is not only an additional part of feedstocks, but it is actively produced for further application in industrial processes. Potential acetate sources range from syngas fermentation to CO_2_ fixation via microbial electrosynthesis [27,28,29,30,31,32].

As a result, the implementation of acetate as a novel substrate in industrial biotechnology has been attempted in recent years and is showing promising results on the small scale. Recent achievements suggest that insights into the acetate metabolism of different microbial species combined with metabolic engineering of industrial host organisms seem to be the way towards sustainable production of value-added chemicals [33,34,35,36,37].

Even though a wide variety of microorganisms are capable of growing on and metabolizing acetate, the focus for further process and strain development is *E. coli*. Therefore, this review will give an overview of acetate metabolism of different species and will then focus on acetate utilization in *E. coli.* We will present products that have already been successfully produced, highlight energetic aspects of growth on acetate, and discuss challenges that need to be overcome by metabolic and process engineering.

## 2. Acetate Metabolism in Different Organisms

The ability to use acetate as a carbon source is present in numerous microorganisms ranging from eukaryotes to bacteria. In the latter, growth on acetate is possible across different phyla. Next to proteobacteria, which include *E. coli*, *Vibrio natriegens*, *Pseudomonas aeruginosa,* and the purple non-sulfur bacterium *Rhodobacter capsulatus*, members of the phylum of firmicutes (e.g., *Clostridium kluyveri*), cyanobacteria (e.g., *Synechocystis* sp. PCC6803) and sulfate-reducing bacteria (which are not part of one phylum but several different) have exhibited growth on acetate [38,39,40,41,42,43]. Even in the kingdom of Plantae, green algae of the phylum chlorophyte (*Chlamydomonas reinhardtii* and *Chlorella sorokiniana*) can use acetate as a carbon source [44,45]. Whether growth occurs aerobically or anaerobically can vary between all the different acetate users.

When acetate constitutes the sole carbon source, the central energy metabolism (generation of ATP), the redox balance of the cells (NADH/NAD^+^ and NADPH/NADP^+^), and the formation of precursors for other metabolites (e.g., amino acids) are distinctly different compared to sugar fermentation. As these factors have a major influence on how well cells grow and produce metabolites, we will focus our overview of the acetate metabolism in different organisms in these areas.

### 2.1. The Aerobic Prokaryotic Acetate Metabolism

Over the last few years, prokaryotic acetate metabolism has become a more researched topic [46,47,48,49,50]. The increased investigation has strongly focused on *E. coli* because it is the organism of choice to test the feasibility of using acetate-based feedstocks. Subsequently, studies regarding uptake and metabolic engineering to further maximize its possibilities in *E. coli* have been published [47,51,52,53,54].

In addition to the extensive research on *E. coli*, other prokaryotic organisms have a similar potential for growth on acetate. Relevant studies include *V. natriegens*, which is an emerging microbial host due to its fast growth and high capacity for substrate uptake, and *P. aeruginosa*, an opportunistic human pathogen with the additional ability to grow on ethanol [39,48,49,55,56,57,58,59]. Different reports on their specific growth rates were summarized in Table 1.

Although the central carbon metabolism of *V. natriegens* and *P. aeruginosa* has not been studied as extensively as the one of *E. coli*, it can be assumed that the metabolism works similarly [48,49,62].

**Figure 1:**
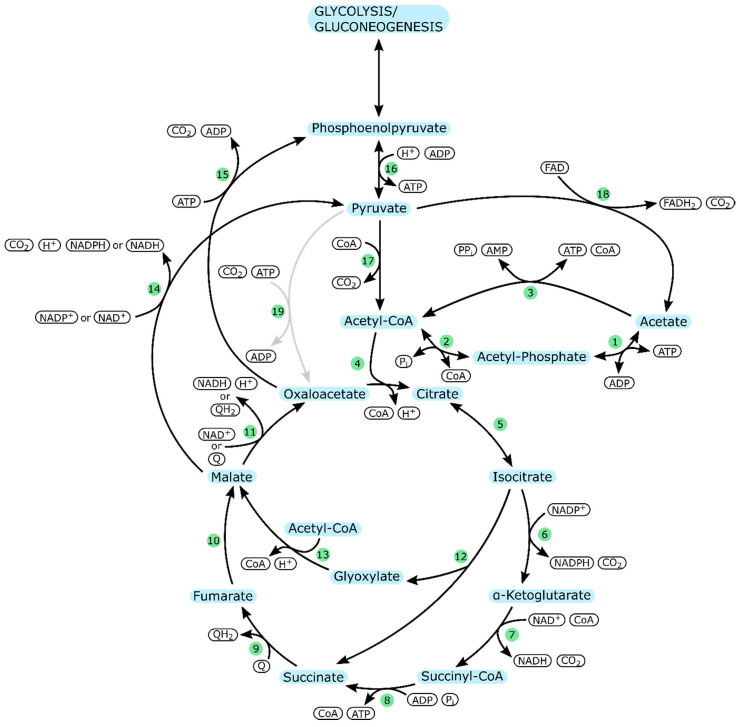
Acetate metabolism in *E. coli*, *V. natriegens*, and *P. aeruginosa*; Arrows indicate the preferred direction of reactions. Gray arrows indicate reactions only present in *P. aeruginosa*. Enzymes are represented by numbers in green circles (cf. Table 2).

Therefore, Figure 1 shows an overview of the central carbon metabolism focusing on acetate catabolism in *E. coli*, *V. natriegens,* and *P. aeruginosa*. The respective enzymes involved are listed in Table 2. Since *E. coli* is the most extensively studied of the three organisms, the following description will be based on knowledge from the *E. coli* metabolism.

As illustrated in Figure 1, acetate can be taken up via two reversible reactions catalyzed by acetate kinase (AckA) and phosphotransacetylase (Pta), which results in the formation of ADP and acetyl-CoA. A second pathway is an irreversible reaction mediated by acetyl-CoA synthetase (Acs), which consumes more energy than the alternative pathway and yields AMP and acetyl-CoA.

For *E. coli,* it has been shown that both pathways are necessary for complete uptake of acetate as without *ackA* acetate can only be taken up to a lower concentration limit of 3.5 mM, and without *pta* and *acs* acetate uptake decreases [63]. However, data from different studies suggest that the *acs*-pathway is the main route for acetate uptake at low acetate concentrations, while assimilation at high concentrations predominantly takes place via AckA and Pta [54,63]. Under certain conditions, it is also possible that the presence of both pathways is the cause of a futile cycle of simultaneous acetate consumption and secretion [54].

Newly formed acetyl-CoA can enter the fatty acid metabolism or the TCA cycle. Since acetate is a low-carbon substrate, the cells attempt to conserve carbon by increasing flux towards the glyoxylate-shunt. Otherwise, CO_2_ would be lost in the reactions of the TCA cycle which would render growth on acetate impossible [38,64,65,66].

Another important issue is the generation and regeneration of energy and reduction equivalents. NAD^+^ and NADP^+^ can only be reduced via the reactions of the TCA cycle. Similarly, ATP is mainly generated via oxidative phosphorylation. Eventually, acetyl-CoA flux through the TCA cycle and the glyoxylate shunt yields oxaloacetate, from which gluconeogenesis may start [67].

### 2.2. The Aerobic Eukaryotic Acetate Metabolism

Acetate as a carbon source has also been investigated for eukaryotic organisms. However, even though frequently used laboratory yeasts like *Saccharomyces cerevisiae* and *Pichia pastoris* can utilize acetate in theory, it is more likely to cause problems in their various industrial applications. Acetate has a strong inhibitory effect on yeasts with critical concentrations as low as 40 mM for *P. pastoris* [68]. *S. cerevisiae* has been reported to be slightly more tolerant, but especially in the context of ethanol production from lignocellulosic hydrolysates, acetate is frequently named as an inhibitor of cell growth and the cause for a reduced ethanol yield [69,70,71,72,73,74].

In contrast to that, *Yarrowia lipolytica* is another model yeast with distinctly different properties. Deriving its biotechnological importance from the ability to excrete lipases, produce citric acid and accumulate lipids, this oleaginous yeast is also capable of growing on acetate [75,76].

**Figure 2:**
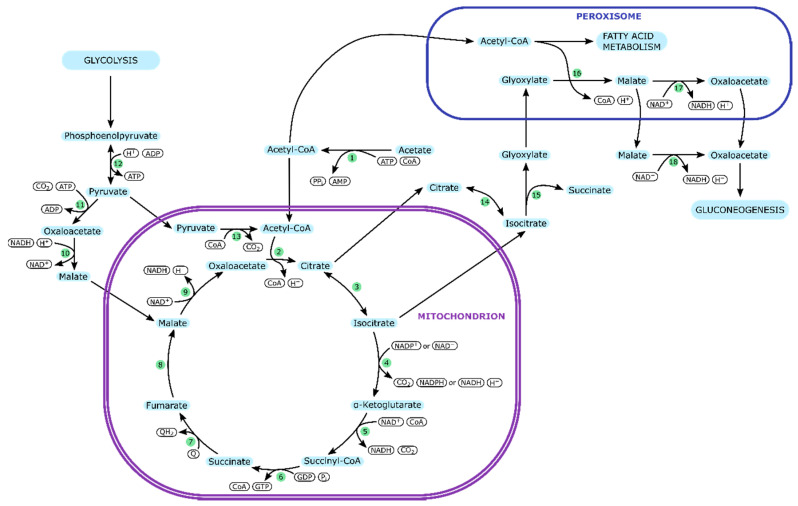
Acetate metabolism in *Y. lipolytica*. Arrows indicate the preferred direction of reactions. Enzymes are represented by numbers in green circles (cf. Table 3).

Figure 2 shows the acetate metabolism in *Y. lipolytica*. The enzymes involved are listed in Table 3.

Because *Y. lipolytica* is a yeast, its acetate metabolism presents itself rather different from *E. coli* and similar bacteria. Although the pathways employed in acetate metabolism are similar to *E. coli*, e.g., also in *Y. lipolytica* the glyoxylate shunt enables growth on acetate as sole carbon source, the reactions of the citric acid cycle and the glyoxylate cycle are confined to different organelles [77].

When assimilated, acetyl-CoA may enter the TCA cycle in the mitochondria, or it may be transported into the peroxisome to participate in the fatty acid metabolism or the glyoxylate cycle. The enzymes of the glyoxylate cycle, are distributed between the cytoplasm and the peroxisome [77]. Citrate is converted to isocitrate in the mitochondria and split into succinate and glyoxylate in the cytoplasm. The condensation of glyoxylate and acetyl-CoA occurs in the peroxisome, yielding malate. Whether malate is oxidized to oxaloacetate in the peroxisome or the cytoplasm is determined by alternative splicing of the gene for malate dehydrogenase, which leads to the formation of either a cytoplasmic or a peroxisomal enzyme. Oxaloacetate can be further metabolized to pyruvate, initiating gluconeogenesis [76,78].

The distribution of these essential reactions across several organelles requires a complex network of transporters, which have been omitted from this description for reasons of simplicity.

### 2.3. The Anaerobic Prokaryotic Acetate Metabolism

Under anaerobic conditions, *E. coli* grown on glucose usually excretes acetate together with other fermentation products (e.g., ethanol and lactate) and uses nitrate or fumarate as electron acceptors [1,79]. Only recently it was rediscovered that the addition of formate as co-substrate results in anaerobic growth mediated by pyruvate-formate lyase, where the glyoxylate cycle is at least partially active [80,81].

Other anaerobic microorganisms exhibit a vastly different metabolism. Lacking oxygen as an electron acceptor, they use a wide range of alternative electron acceptors, including hydrogen, sulfur, sulfate and nitrite [41,82,83,84,85]. This diversity is also reflected in the acetate-utilization pathways. For this review, two examples were chosen to present the acetate metabolism of anaerobic prokaryotes: *C. kluyveri* and the group of sulfate-reducing bacteria.

#### 2.3.1. *Clostridium kluyveri*

**Figure 3:**
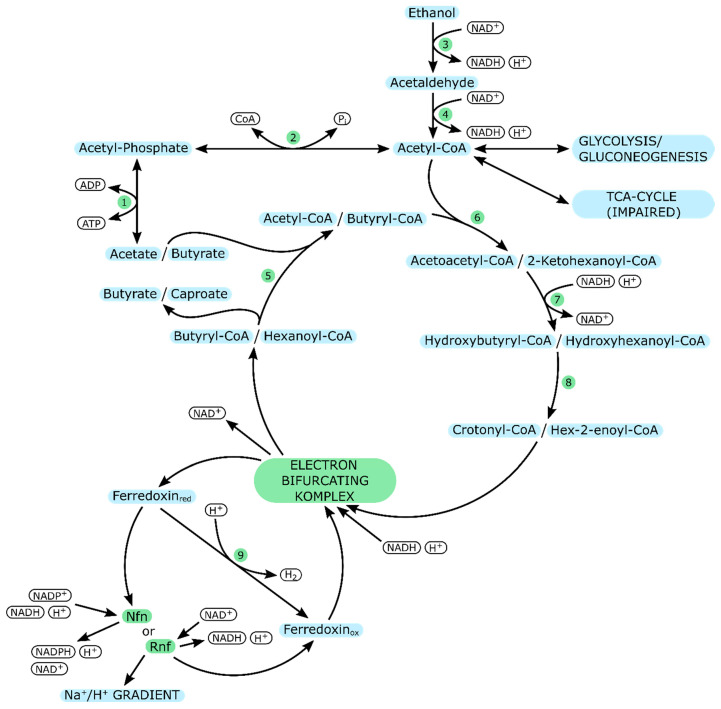
Acetate and ethanol utilization in *C. kluyveri*; Arrows indicate the preferred direction of reactions. Enzymes are represented by numbers in green circles (cf. Table 4).

*C. kluyveri* has gained biotechnological importance due to its ability to produce medium-chain organic acids from acetate and ethanol [86,87]. The cells can accomplish that by implementing a reverse β-oxidation, outlined in Figure 3.

In this pathway, ethanol acts as the electron donor, whereas acetate assumes the role of an electron acceptor [88]. Acetyl-CoA derived from ethanol is condensed with acetyl-CoA derived from acetate to form acetoacetyl-CoA and subsequently hydroxybutyryl-CoA and crotonyl-CoA [89]. The conversion of crotonyl-CoA to butyryl-CoA is catalyzed by an electron bifurcating butyryl-CoA dehydrogenase complex [90]. This enzyme is ferredoxin-dependent. Reduced ferredoxin can be reoxidized in different ways. The first is via a ferredoxin-dependent hydrogenase, forming H_2_ in the process [88]. The second way depends on substrate concentration. At low concentrations (about 1 mM), an NADH-dependent reduced ferredoxin:NADP oxidoreductase oxidizes ferredoxin and yields NADPH, at high substrate concentrations (about 1 M), a ferredoxin-NAD reductase complex builds up an electrochemical gradient essential for transport coupled phosphorylation [88].

Once the conversion of crotonyl-CoA to butyryl-CoA is complete, butyryl-CoA can either leave the reverse β-oxidation cycle or be elongated further to caproate in another round [36].

The ability of *C. kluyveri* to produce medium-chain organic acids in this way is frequently utilized in microbial consortia, where the respective substrates are produced by other microorganisms [82,86,87].

#### 2.3.2. Sulfate-Reducing Bacteria

There are two main groups of sulfate-reducing bacteria. One group can metabolize organic compounds incompletely and produces acetate while the other one can completely convert organic compounds to CO_2_ [43]. The latter group will be presented in this review.

**Figure 4:**
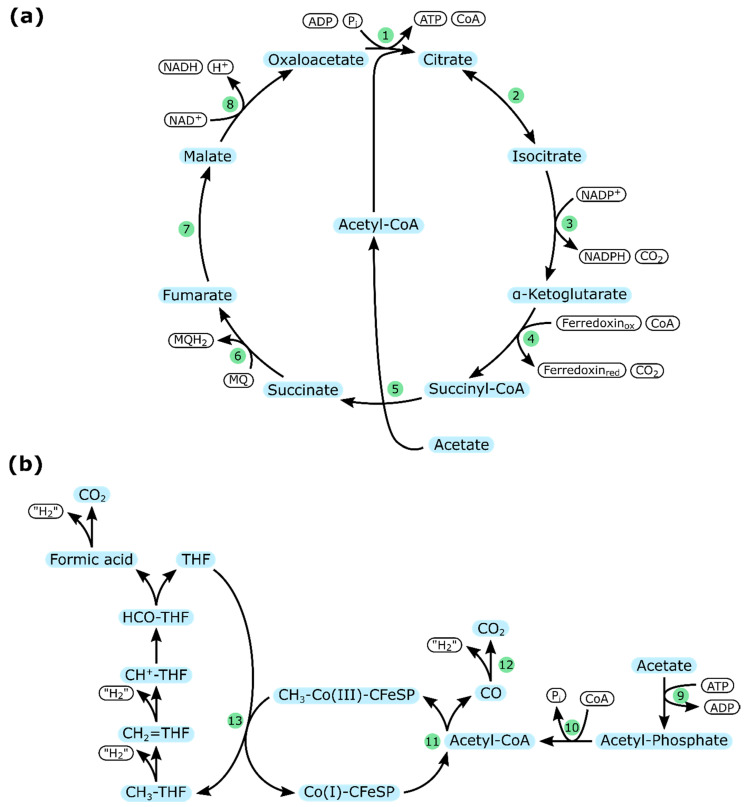
Metabolic adaptations of sulfate-reducing bacteria to utilize acetate: (**a**) modified citric acid cycle as used by *Desulfobacter postgatei*; (**b**) reverse Wool–Ljungdahl pathway as employed by organisms like *Desulfobacter autotrophicum*; arrows indicate the preferred direction of reactions. Enzymes are represented by numbers in green circles (cf. Table 5). “H_2_” indicates the generation of two protons and two electrons, which may result in the formation of reduced ferredoxin, NADH, or NADPH.

Depending on the organism, different metabolic adaptations (Figure 4) enable the utilization of acetate as a carbon source.

In some sulfate-reducing bacteria, like *Desulfobacter postgatei*, acetate is completely metabolized to CO_2_ via the citric acid cycle (Figure 4a). Acetyl-CoA is formed from acetate by a succinyl-CoA:acetate-CoA transferase. The entry point of acetyl-CoA to the TCA cycle is slightly different than in *E. coli* or *Y. lipolytica* because the formation of citrate is catalyzed by an ATP-citrate-lyase. Electrons originating from the TCA cycle are transported towards sulfate as the terminal electron acceptor [91].

Other sulfate-reducing bacteria, like *Desulfobacter autotrophicum*, employ a reverse Wood-Ljungdahl pathway to grow on acetate (Figure 4b). However, since the conversion to CO_2_ is endergonic, the reactions are coupled to the thermodynamically favorable reduction of sulfate [92,93].

In natural environments, sulfate-reducing bacteria must often compete with methanogens for available acetate. Regardless of the pathway used for the metabolism, it has been shown multiple times that sulfate-reducing bacteria can outcompete methanogens if enough sulfate is available [94,95]. As a result, the sulfate reduction rate influences the rate of methane production, which can have a significant impact on the atmospheric carbon emission of sludges, slurries, and estuary sediments [96].

## 3. Products from Acetate

### 3.1. Acetate-Based Production in Different Microorganisms

The attempt to produce value-added products from acetate has been made in several different microorganisms. Some examples are shown in Table 6, mainly comprising substitutes for fossil fuels and platform chemicals.

As can be seen, the range of microorganisms for the production of lipids is diverse, including bacteria (*Rhodobacter* sp. KKU-PS1), yeasts and fungi (*Y. lipolytica* and *Cryptococcus curvatus*), and even microalgae (*Auraniochytrium limacinum* SR21) [97,98,99,100].

Polyhydroxyalkanoates (PHAs) and especially polyhydroxybutyrate are another important class of value-added chemicals. Their production from acetate has been reported from the phototrophic bacterium *Dinoroseobacter* sp. JL1447, the cyanobacterium *Synechocystis* PCC 6803, and the bacteria *Pseudomonas putida* KT2440 and *Aeromonas hydrophilia* [37,101,102,103]. For *Dinoroseobacter* sp. JL1447, acetate as carbon source was even found to sustain a higher PHA production rate than others like glucose, glutamate, pyruvate, or citrate [101].

Other chemicals such as histidine, caproate, malic acid, molecular hydrogen, and methane/biogas have a narrower range of hosts for production from acetate (cf. Table 6) [36,105,106,107,109,110,111]. The generation of isotope-labeled L-glutamate or electricity via microbial fuel cells seem to be special cases and constitute less common ways to upgrade acetate into value-added products [108,112].

Most organisms listed in Table 6 were subject to little or no extensive genetic manipulation and generally constitute natural producers of these particular products. Genetic engineering was only required in some cases, where auxotrophic mutants were created, acetate uptake had to be enabled or product pathways were introduced [103,104,105].

However, coupling the consumption of acetate to the production of value-added chemicals in their natural producers can be challenging, especially if genetic or metabolic information on the organism is incomplete, the cultivation conditions are challenging or tools and procedures for genetic manipulation are lacking. Thus, the range of efficient, industrially applicable producers as well as products in these mostly non-model organisms is somewhat limited.

### 3.2. Acetate-Based Production in E. coli

In contrast, *E. coli,* as a well-studied model organism, has become the focal point for the production of acetate. While some chemicals, like lipids and gases, are not commonly produced in *E. coli*, small organic acids, alcohols, and amino acids derived from metabolic intermediates can readily be generated. Other possible products include polyhydroxybutyrate, phloroglucinol, and β-caryophyllene (Table 7).

This wider range in *E. coli* can be attributed to the availability of tools for extensive genetic and metabolic engineering. For all the reported products, at least one heterologous gene was necessary and often even a whole heterologous pathway [33,113,114].

**Table 7:** Products from acetate as carbon source in *E. coli*. Maximum reported yields under aerobic conditions were listed if available. Theoretical yields were calculated as energy-balanced and non-energy-balanced. Non-energy-balanced: Only reactions from educt to product were considered and balanced according to carbon stoichiometry, no cofactor- or redox-balance. Energy-balanced: Reactions from educt to product and reactions replenishing the consumed energy in the form of ATP were considered. For calculations see text and Supplementary Materials. Reported yields higher than the energy-balanced maximum theoretical yield can be attributed to complex media additives like yeast extract.

Product	Energy Balanced	Theoretical Yield		Theoretical Carbon Yield	Max. Reported Yield (Aerobic)	Reference
	[yes/no]	[mol/mol]	[g/g]	[%]		
Acetoin	No	0.25	0.37	50	0.09 g/g ^1^	[115]
	Yes	0.29	0.44	58		
Acetone	No	0.50	0.49	75	0.29 mol/mol	[113]
	Yes	0.39	0.38	58		
N-Acetylglutamate	No	0.25	0.80	88	n.a.	[116]
	Yes	0.22	0.70	77		
2,3-Butanediol	No	0.25	0.38	50	0.09 g/g ^1^	[115]
	Yes	0.27	0.41	54		
β-caryophyllene	No	0.11	0.38	83	0.02 g/g	[33]
	Yes	0.07	0.24	51		
Glycolate	No	n.a.	n.a.	n.a.	0.58 g/g	[117]
	Yes	1.00	1.27	100		
3-Hydroxybutyrate (PHB)	No	0.50	0.87	100	0.25 g/g	[118]
	Yes	0.35	0.61	70		
3-Hydroxypropionic acid	No	1.00	1.51	150	0.30 g/g	[119]
	Yes	0.50	0.75	75		
Isobutanol	No	n.a.	n.a.	n.a.	0.025 mol/mol	[120]
	Yes	0.25	0.31	50		
Isopropanol	No	0.50	0.51	75	0.56 mol/mol	[114]
	Yes	0.35	0.36	53		
Itaconic acid	No	n.a.	n.a.	n.a.	0.07 mol/mol	[60]
	Yes	0.33	0.73	83		
Mevalonate	No	0.33	0.84	100	0.30 g/g	[121]
	Yes	0.23	0.57	68		
MNEI	Single chain of the sweet plant protein monellin; no stoichiometric calculation of yields possible					[122]
Phloroglucinol	No	0.33	0.71	100	0.18 g/g	[123,124]
	Yes	0.24	0.52	72		
Succinate	No	0.50	1.00	100	0.46 mol/mol	[125,126,127]
	Yes	0.44	0.88	88		
Tyrosine	No	n.a.	n.a.	n.a.	0.04 g/g	[128]
	Yes	0.13	0.38	56		

^1^ The yield given here is actually for 2,3-butanediol and acetoin together.

### 3.3. Comparability between Products from Acetate in E. coli

Since most efforts to use acetate as the carbon source for value-added products originate from the goal to establish a biobased production and reduce CO_2_ emissions, efficient carbon conversion is paramount [114,123]. This efficiency is reflected in the yields, especially the carbon yield.

To enable an evaluation of the efficiency of a given process, the process yields need to be compared to the maximum theoretical yields (MTYs). However, calculating the MTYs can be challenging and there seem to be two general ways by which they are obtained.

In some instances, MTYs are calculated by considering only the carbon stoichiometry of reactions necessary to convert the carbon source to the product, regardless of the amounts of ATP, NADH, or NADPH required for the generation of the target molecule (not energy-balanced) [113,114,125].

In other instances, it is taken into account that for the continuous supply of ATP, NADH, and NADPH, additional molecules of the carbon source need to be taken up and metabolized, which might also result in the release of CO_2_ [60,119]. In these cases, the given MTYs can be considered redox and energy balanced.

In a few cases (glycolate, itaconic acid, isobutanol, and tyrosine) the production pathways are inherently energy-balanced. In these instances, follow-up reactions of the acetate molecules that are required for the carbon balance compensate for the energy consumption of acetate uptake and product generation. As an example, the energy expense for the formation of itaconic acid is covered by acetyl-CoA entering the TCA and glyoxylate cycle, where NADH, FADH, and subsequently ATP is regenerated [60]. Therefore, no further consideration of energy requirements is necessary.

This diversity in the calculations complicates comparability as shown in Table 7. To further illustrate the impact of these different calculation methods, we present them for two examples: acetone and itaconic acid. For the calculations, the yield of ATP per oxygen (phosphate/oxygen or p/o ratio) was assumed to be two, and ATP equivalents from FADH, NADH, and NADPH to be one, two, and two, respectively [9,129]. We discuss these assumptions in more detail below.

If acetone is produced from acetate, two molecules of acetate need to be taken up. The resulting two acetyl-CoA are subsequently condensed to acetoacetyl-CoA and react further to acetoacetate. In the last step of the acetone pathway, acetoacetate is converted to acetone by decarboxylation. Considering only these reaction steps, the carbon-balanced formation of acetone from acetate may be described by Equation (1). Molecules with no impact on the carbon or energy balance were omitted.

2 acetate → 1 acetone + 1 CO_2_ (1)

As a result, the maximum theoretical yield of acetone from acetate is 0.50 mol/mol or 0.49 g/g and the theoretical carbon yield amounts to 75%.

However, it should also be taken into account that the acetate uptake consumes ATP. Here, we assumed that acetate was taken up via the energetically more expensive acetyl-CoA synthetase, which requires two ATP per acetate. Therefore, Equation (1) needs to be amended towards Equation (2).

2 acetate + 4 ATP → 1 acetone + 1 CO_2_ (2)

The consumed ATP may be replenished if another molecule of acetate is taken up and enters the TCA cycle. With the assumptions on ATP equivalents made above, the two ATP for the uptake are balanced out by 9 ATP gained from TCA cycle associated reactions, leaving a surplus of 7 ATP. This is enough to balance the ATP-requirement for the acetone synthesis, however, the reactions of the TCA cycle also produce two additional molecules of CO_2_. As a result, we can formulate Equation (3).

3 acetate → 1 acetone + 3 CO_2_ + 3 ATP (3)

To finalize the equation, we want to eliminate the ATP. Since we have established above that one molecule of acetate equals 7 ATP and two CO_2_, the final energy balanced reaction is given in Equation (4).

18 acetate → 7 acetone + 15 CO_2_ (4)

The new energy-balanced yield constitutes 0.39 mol/mol or 0.38 g/g and the theoretical carbon yield is 58%.

Next, we examine the production of itaconic acid. First of all, it requires the uptake of three molecules of acetate. Two of the three resulting acetyl-CoA enter the TCA cycle and together with two molecules of oxaloacetate, they form two molecules of citrate and subsequently isocitrate. From here, one isocitrate can react to itaconic acid and CO_2_, while the other one follows the reactions of the glyoxylate shunt. In the glyoxylate shunt, the third molecule of acetyl-CoA needs to condense with glyoxylate to form malate. In the end, the reactions replenish the two molecules of oxaloacetate that were required at the start of the TCA cycle. Therefore, the carbon-balanced synthesis of itaconic acid can be given in Equation (5).

3 acetate → 1 itaconic acid + 1 CO_2_ (5)

If we examine the energy requirement, we can see that 6 ATP are needed for the uptake of 3 molecules of acetate. However, the subsequent reactions of the glyoxylate shunt generate two NADH and two FADH, which amounts to a total of 6 ATP. As a result, Equation (5) is inherently energy-balanced with the yield from acetate constituting 0.33 mol/mol or 0.73 g/g and the theoretical carbon yield 83%.

The different calculation strategies can have a considerable impact. Firstly, it should be noted that no biological system can surpass an energy balanced yield, meaning if acetate is used as the sole carbon source, the MTY of acetone can never be above 0.39 mol/mol. Therefore, the highest reported yield for acetone (0.29 mol/mol) is already relatively close to the biological maximum [113]. This should always be kept in mind if measures to improve process yields are taken.

Another issue might be the comparison of two products from acetate, especially, if the maximum theoretical carbon yield (MTCY) is involved as we outlined above for the two metabolites acetone and itaconic acid. If a non-energy-balanced calculation is applied, it might seem that acetone and itaconic acid have similar MTCYs, 75% and 83%, respectively. However, this is only because the pathway for itaconic acid is inherently energy-balanced while the one for acetone is not [60,113]. If an energy-balanced calculation method is applied, acetone has a much lower MTCY (58%) than itaconic acid (83%).

Therefore, to avoid these pitfalls, it would be advisable to work with energy-balanced MTYs.

Whether the MTY is energy-balanced or not, might not be the only challenge, though. If an energy-balanced approach is chosen, there is still the issue of how to weigh ATP, NADH, NADPH, and FADH against each other. In aerobic cultivations with acetate as the sole carbon source ATP is mainly gained via oxidative phosphorylation. The yield of ATP per oxygen is given by the phosphate/oxygen (p/o) ratio, which in turn depends on the electron transport efficiency and the H^+^/ATP-ratio [130,131].

In *E. coli*, different kinds of dehydrogenases with varying H^+^/e^−^-ratios have been identified. They are responsible for proton translocation across the membrane. The ratio of H^+^ to ATP is also a topic for debate, with reports ranging from 2 to 4 resulting in p/o-ratios between 1,5 and 3 [131,132,133]. Therefore, finding a conclusive way to calculate the ATP equivalents of NADH, NADPH and FADH is difficult and a compromise has to be made [79,134].

## 4. Energetic Comparison between Acetate, Glycerol, and Glucose as Carbon Sources in *E. coli* under Aerobic Conditions

Based on the assumptions made above, acetate has an MTY of 7 ATP. Two ATP are needed for acetate uptake (irreversible uptake via acetyl-CoA synthase was assumed) while 9 ATP are generated during the oxidation of acetyl-CoA in the TCA cycle. Compared to other carbon sources like glycerol and glucose, the ATP yield of acetate is rather low.

**Figure 5:**
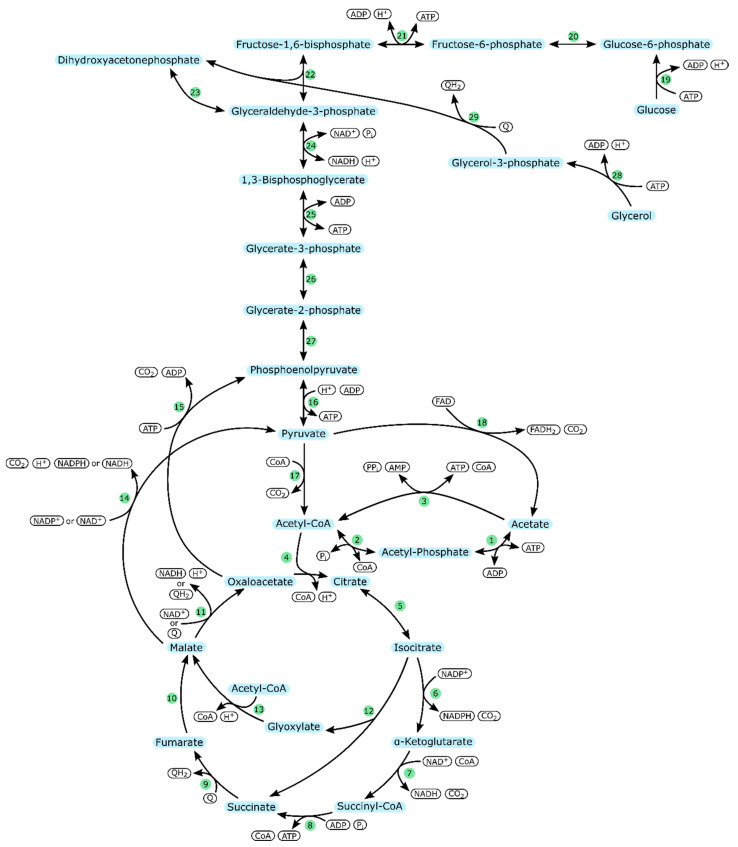
Central carbon metabolism of *E. coli* for acetate, glucose, and glycerol as carbon sources under aerobic conditions. A list of the depicted enzymes is given in Table 8.

As can be inferred from Figure 5 (cf. Table 8), other commonly used carbon sources in *E. coli* like glucose or glycerol are more favorable in terms of ATP yield under aerobic conditions. For glucose uptake and conversion to fructose-1,6-bisphosphate requires 2 ATP. Then, the conversion of two glyceraldehyde-3-phosphate to two 1,3-bisphosphoglycerate generates 2 NADH, and 2 ATP are recovered from two 1,3-bisphosphoglycerate to glycerate-3-phosphate. The reactions of 2 phosphoenolpyruvate to pyruvate finally yield two additional ATP. The remaining 18 ATP originate from 2 acetyl-CoA entering the TCA cycle according to the equivalence assumptions for the conversion of FADH, NADH, and NADPH to ATP. In total, 24 ATP are gained from glucose this way.

For the uptake of glycerol via glycerol-3-phosphate, one ATP is needed. Two ATP equivalents are generated in the next step, yielding dihydroxyacetone phosphate. The subsequent reactions towards pyruvate and the TCA cycle are the same as with glucose. However, this time only one 3C-unit is processed. This leads to an overall ATP gain of 14 ATP per glycerol.

In terms of energy content, the main challenge concerning acetate is the carbon’s high oxidation state. By using glucose or glycerol some energy equivalents are generated independently of the reactions of the TCA cycle. As portrayed in Figure 5, ATP can be gained directly by substrate-level phosphorylation or indirectly via the oxidation of glycerol to glyceraldehyde and further to glycerate. Acetate as a carbon source cannot provide this extra energy supply since ATP can only be gained from oxidative phosphorylation in combination with the TCA cycle reactions.

Moreover, since most acetyl-CoA has to enter the TCA cycle for sufficient energy supply, the production of sugars can only be fueled by activation of the glyoxylate shunt [135]. Even though carbon can be diverted towards anabolic reactions in this way (e.g., towards the pentose phosphate pathway), the generation of glucose or ribose still requires some additional energy.

Another important aspect of the growth of any carbon source is the availability of cofactors. While the oxidation of NADH and NADPH can be used to build up a proton gradient across the membrane to generate ATP, there are also several other vital reactions in the cell, which require reduction equivalents [136,137,138]. As a result, not only the demand for energy equivalents must be kept in mind, but also whether they are available as NADH, NADPH, or ATP. Addressing this issue, cofactor engineering has gained importance to improve product yields [114,120,139,140,141].

However, despite all the challenges that acetate entails energetically, one of its advantages might be that its entry point into the metabolism (acetyl-CoA) is very close to the potential products’ branching points from the central carbon metabolism. With additional metabolic engineering, this “metabolic proximity” could allow for tight regulation of production.

## 5. Engineering of *E. coli* to Optimize Productivity on Acetate

Engineering *E. coli* for better productivity, yields, and product titers on acetate can be conducted on two main levels.

The first level is metabolic engineering, which comprises several major aspects. Most importantly, it enables the introduction of non-native pathways into the *E. coli* host strains, which is the main prerequisite for acetate-based production [114,120,125]. Moreover, metabolic engineering may improve acetate uptake and tolerance [35,123]. Another important aspect of metabolic engineering is co-factor engineering, especially redox-cofactor (NADH and NADPH) engineering. NADH and NADPH are not only essential for some reactions in the synthetic product pathways (predominantly for alcohols) but also affect the cellular redox homeostasis. As a result, the availability of NADH and NADPH can substantially influence the growth and metabolism of the cells [142].

The second level of engineering is process engineering. As process conditions like pH may substantially affect growth and metabolism on acetate, tight regulation is often necessary [63]. In other instances, the in situ removal of toxic products may be required to prevent cell death and still achieve a high product recovery [113,143]. Finally, coupling acetate production processes to acetate utilization processes could lead to the implementation of continuous production chains from sustainable acetate to more complex chemicals [114].

### 5.1. Metabolic Engineering

#### 5.1.1. Pathway Engineering

**Figure 6:**
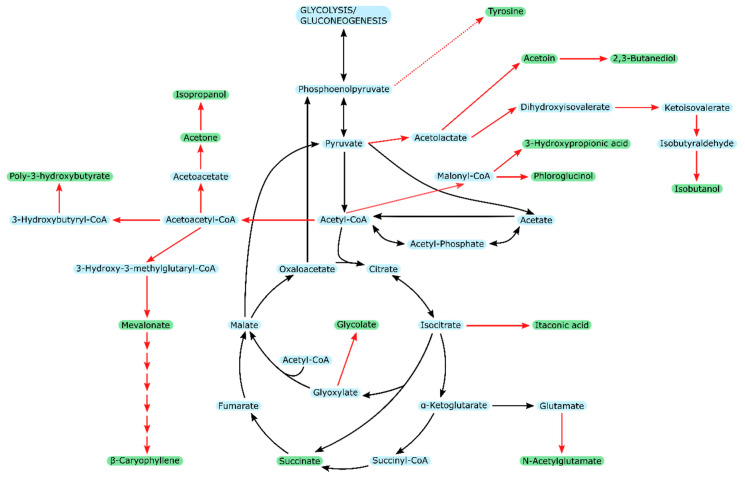
Overview of all products from acetate in *E. coli* listed in Table 7 and their ties into the central carbon metabolism. Red arrows indicate engineered pathways; green labels constitute end-products of engineered pathways.

Naturally, the first aspect of metabolic engineering of *E. coli* concerning the production of value-added chemicals from acetate is the introduction of the metabolic pathways leading to the desired product. Figure 6 shows an overview of the numerous metabolites that have already been demonstrated (cf. Table 7).

The pathways branching from the central carbon metabolism can be constructed in different ways. In the case of tyrosine, the pathway is natural to the *E. coli* host strain, but the expression levels of the involved enzymes were optimized to favor tyrosine production [128,144]. For the production of acetone, N-acetylglutamate, PHB, 3-hydroxypropionic acid, itaconic acid, mevalonate, and phloroglucinol the pathways contained one gene derived from a different organism than *E. coli*, resulting in the construction of expression cassettes containing extra copies of the *E. coli* genes and the heterologous ones [60,113,116,118,119,121,123]. Truly synthetic pathways incorporating genes from several organisms were employed for the production of β-caryophyllene, isobutanol, isopropanol, and succinate [33,114,120,125,145]. Appropriate expression cassettes and full pathways are typically assembled in plasmids, which can then be transformed into the desired *E. coli* host strain [33,60,113,114,116,117,118,119,120,121,123,125,126,127,128].

In some cases, additional gene deletions are necessary to disable unfavorable pathways, in other instances pathway genes are deleted and reintroduced into the cells under a promoter which allows expression control [60,113,114,117,125,126,127,128].

The types of deletions and modifications depend on the branching point from the central carbon metabolism. However, the starting point for all products is acetate uptake. Therefore, the strategy of enhancing acetate uptake to improve productivity has been employed with products from different branching points, such as acetone, isopropanol, tyrosine, succinate, and glycolate [113,114,117,125,127,128].

As for the question of how to increase acetate uptake, there are two approaches to tackle this challenge. As in *E. coli* acetate can either be taken up via the reversible *ackA/pta* pathway or the irreversible *acs*-mediated way, these are the two points of action [54,63]. Some works have successfully overexpressed the *ackA/pta* pathway to improve acetate uptake and production of acetone, isopropanol, succinate, or glycolate [113,114,117,125]. Others have also successfully increased the expression of *acs* to enhance acetate uptake with products like succinate, phloroglucinol, tyrosine, or mevalonate [121,123,127,128].

It is extremely difficult to tell if either option is generally better or worse than the other one. Arguments for choosing the *ackA/pta* pathway usually include the lower energy requirement compared to uptake via acetyl-CoA synthetase (Acs) and its prevalence on high acetate concentrations [113,117,125]. Contrary, it is argued that Acs has a higher substrate affinity and its reaction is not reversible [127].

In the case of itaconic acid, it has been reported that overexpression of the *ackA/pta* pathway instead of *acs* was detrimental to cell growth and did not improve production [60]. Similarly, the synthesis of PHB did not benefit from *acs*-overexpression but rather worked better via enhancing the *ackA/pta* pathway [118]. These findings are peculiar as they are contrasted by reports of successful overexpression of the *ackA/pta* pathway which did increase acetate uptake and thereby the product titer and yield [113]. The reason for this discrepancy is as of yet unknown and requires further investigation. However, the type of product or the different genetic and metabolic manipulations might play a role since it has been shown that acetyl-phosphate, the intermediate of the *ackA/pta* pathway, plays an important regulatory role in the cells. Acetyl-phosphate affects protein acetylation and in extension glucose metabolism and acetyl-CoA pools as well as the phosphorylation of numerous response regulators like CheY, PhoB, OmpR, and CpxR [146,147]. These regulators are involved in chemotaxis and the responses to phosphate starvation, osmotic stress, and envelope stress [148,149,150,151]. As acetyl-phosphate exhibits such a diverse influence it has been theorized that it might affect various acetate-based products differently [51,54].

Apart from acetate uptake, with products branching from pyruvate, it seems that the most efficient way to increase the yield is enhancing pyruvate availability. It has been shown that the overexpression of *maeB* (conversion of malate to pyruvate) and *pckA* (conversion of oxaloacetate to phosphoenolpyruvate) increases isobutanol production [120]. The addition of asparagine/aspartate, which may enhance the available oxaloacetate pool, has also proven beneficial to produce the pyruvate-derived products 2,3-butanediol and acetoin from acetate [115].

For phosphoenolpyruvate-derived chemicals, the situation is less clear, since the only reported product is tyrosine. In this case, the expression of the glyoxylate cycle enzyme AceA was altered. With this strategy, it was possible to find a balance between carbon conservation via the glyoxylate shunt and energy generation via the TCA cycle thereby increasing the tyrosine yield [128].

Some products branching from acetyl-CoA seem to benefit from enhancing the glyoxylate shunt. Flux through the glyoxylate shunt can either be increased by direct upregulation (e.g., deletion of IclR, a transcriptional repressor of the glyoxylate shunt) or by blocking the TCA cycle via deletion of isocitrate dehydrogenase [113,119]. In the case of phloroglucinol synthesis from acetate, a knockout of the endogenous citrate synthase (*gltA*) improved productivity [124]. In a further study, it was suggested that controlling or altering the activity of citrate synthase could enhance the availability of acetyl-CoA [152]. As an example, the introduction of a toggle switch for citrate synthase was able to successfully improve isopropanol production from glucose [153]. Other approaches for acetyl-CoA derived metabolites are more pathway-specific, like choosing the most efficient genes from different species and appropriate promoters or deal with cofactor engineering [113,114,118].

To successfully produce chemicals derived from the TCA cycle, the reactions leading away from them or their precursors need to be blocked in most cases. For glycolate, any pathway decreasing the glyoxylate pool, except the glycolate production, must be stopped [117]. To obtain succinate, the glyoxylate flux is enhanced to reduce carbon loss (deletion of IclR). Additionally, *sdhAB* is also disabled to prevent flux away from succinate and strains may also be lacking *maeB* and *pckA* to reduce losses of oxaloacetate [125,127].

Next to that, it might also be necessary to actively replenish TCA cycle intermediate pools. For instance, the production of itaconic acid can be improved by enhancing the flux of the glyoxylate shunt. That way, isocitrate as the precursor for itaconic acid is replenished [60].

In conclusion, pathway engineering strategies are highly product dependent and must also be adapted to the branching point from the central carbon metabolism.

#### 5.1.2. Acid/Acetate Tolerance

A repeatedly encountered challenge when using acetate as a carbon source is its potential toxicity to the cells. In concentrations above 5 g/L it can inhibit cell growth substantially, thereby complicating possible production processes [154]. As a result, strategies for improved acetate tolerance are necessary.

The question of how to deal with acetate is not only encountered when it is used as the sole carbon source but also when cells are grown on glucose [8]. In some cases, it has been proven beneficial to eliminate its formation by weakening or removing acetate metabolism [19,21]. However, this is not an option when cells are dependent on acetate for growth and production.

There are several approaches to explain acetate or acid toxicity in general. One of the theories addressing this issue is the uncoupling theory. The reasoning behind this theory is that acetic acid in its protonated form can traverse the cell membrane and dissociate inside the cell. To maintain the proton gradient across the membrane, the resulting proton must be expelled from the cell again, which requires energy (ATP). Subsequently, this energy is not available for growth [15].

A closely related explanation for acid toxicity is anion accumulation. It assumes that when acetic acid diffuses into the cells and dissociates, the intracellular pH decreases. This decrease leads to an accumulation of anions with potentially detrimental osmotic consequences for the cells [14].

To counter excessive acid stress, *E. coli* actually has a number of natural acid tolerance systems, which have been thoroughly reviewed elsewhere [155]. The most prominent of these systems is the glutamate-dependent acid resistance system [156,157].

However, different studies dealing with the production of n-heptanoic and octanoic acid showed that the responsible enzymes are expressed differently in various *E. coli* strains, which is possibly the cause of strain-dependent acid resistance [158,159]. Consequently, one way to improve the tolerance would be to choose a strain that is naturally more resilient.

Acetate tolerance of different *E. coli* strains have already been characterized several times. On defined medium and with an acetate concentration of 85 mM (approx. 5 g/L), *E. coli* C (ATCC8739) was found to have the highest maximum specific growth rate (µ_max_) with 0.41 ± 0.01 h^−1^, followed by *E. coli* W (0.37 ± 0.01 h^−1^). At the same conditions, *E. coli* BL21 and MG1655 exhibited a µ_max_ of 0.30 ± 0.01 h^−1^ and 0.29 ± 0.01 h^−1^ respectively [61]. When grown on acetate with a concentration of 166.5 mM on a defined medium supplemented with 2 g/L yeast extract *E. coli* W reached a µ_max_ of 0.46 h^−1^, while *E. coli* BL21(DE3) and MG1655 grew at 0.36 h^−1^ and 0.23 h^−1^ respectively [60].

The expression and regulation of genes conferring acetate resistance was examined in a study about the cAMP receptor protein (CRP). There, it was discovered that one particular mutation (D138Y) led to an improved growth rate under acetate stress, albeit with a significantly longer lag-phase [154]. Unfortunately, the tested mutant exhibited a downregulated acetate uptake and glyoxylate cycle, which rendered it unfit for growth and production on acetate.

Other studies concerning acid stress in *E. coli* have identified the inhibition of methionine biosynthesis as a cause for reduced growth. When MetE, the first enzyme of the methionine synthesis pathway, is blocked, the toxic intermediate homocysteine accumulates [160]. Accordingly, a modified methionine synthase has been reported to increase acetate tolerance in *E. coli* [161].

Another possibly influencing factor is the intracellular concentration of acetyl-phosphate [13]. Acetyl-phosphate plays an important role in the phosphorylation of two-component response regulators and protein acetylation [51,146,162,163,164]. Therefore, its abundance might be critical for several intracellular processes during growth on acetate [13].

To find further mechanisms for acetate tolerance and to create tolerant strains, adaptive laboratory evolution is a means that can be employed. As it was already successfully used to improve acetate tolerance and growth of *E. coli* under anaerobic conditions on glucose, it can be assumed that similar experiments can also lead to more acetate tolerant *E. coli* under aerobic conditions [165,166].

#### 5.1.3. Product Tolerance

A topic related to acetate tolerance is product tolerance. For acidic products like 3-hydroxypropionic acid, the issue of acid tolerance arises too. While this has been addressed in the production of glycerol, the utilization of an acid-tolerant strain for 3-hydroxypropionic acid has yet to be implemented for acetate as the substrate [119,167]. Contrarily, itaconic acid has already been produced in the more acid-tolerant *E. coli* W strain [60].

Phloroglucinol is another product that has a toxic effect on *E. coli* [124]. To alleviate toxicity, the activation of general stress response genes or the expression of heat shock proteins has been proposed [123]. While these approaches (expression of MarA and GroESL) were successfully applied with glucose as a carbon source, so far none have been adopted for acetate-based production [168,169].

For other products, different solutions to improve tolerance are possible. In the case of isopropanol, adaptive laboratory evolution led to insights on genes involved in alcohol tolerance. The study identified mutations in five genes (*relA*, *marC*, *proQ*, *yfgO*, and *rraA*) that could enhance isopropanol tolerance [170]. Such findings can be used as a basis for further experiments. However, the challenge for alcohol production might be that the cells’ adaption(s) to it may inhibit the synthesis.

#### 5.1.4. Cofactor Engineering

The availability of energy and reduction equivalents is of great importance (see Section 3.3). This is especially the case when a synthetic pathway is applied for production, which might negatively influence the cells’ reduction or energy state.

This issue seems to arise predominantly for the generation of alcohols. Concerns over the NADPH supply have led to the introduction of an additional NADPH regeneration system in the acetate-based production of isobutanol (*fdh* from *Candida boidinii* and *pntAB* from *E. coli*) [120]. The productivity was thereby improved, but contrary to expectations not because of the altered cofactor balance. Instead, the added formate led to an increased pyruvate pool, which benefited the pyruvate derived isobutanol [120].

Contrarily, cofactor engineering was successfully applied to increase the product yield of acetate-based isopropanol. Additional *pntAB* or *nadK* was introduced into the cells and *pntAB* was deemed especially suitable to improve the yield as it replenished NADPH which was consumed by isopropanol production most effectively [114].

Another example is the pathway for mevalonate synthesis which also demands NADPH. In a recent study, the importance of NADPH regeneration for the production of mevalonate was revealed [171]. However, the strategy presented would need to be thoroughly revised for acetate-based processes since it was established for glucose as a carbon source and so far, the implementation for acetate-based production is lacking.

To facilitate cofactor engineering, helpful tools for metabolic engineering have been developed recently. In silico cofactor, balance estimation provides the possibility to evaluate different routes to a target product and allows a more efficient strain design [172].

The emergence of an *E. coli* strain capable of being a platform for testing different enzymatic NADH regeneration systems might also be useful for future metabolic engineering. The strain in question is dependent on the introduction of an NADH regeneration system to grow on acetate. Subsequent determination of the growth rates of cells with different NADH regeneration systems enables the identification of a suitable cofactor regeneration strategy under the applied conditions [141].

### 5.2. Process Engineering

In the case of acetate-based production, process engineering has to deal with a few key factors to ensure stable and reproducible results.

The first issue of special importance seems to be the tight regulation of the pH. Recent results show that the difference between pH 6 and 7 can have a major impact on the expression of acetate metabolism genes [63]. Although the study was conducted with cells grown on glucose-containing media, it can be assumed that the observed effects are equally relevant for cultivations on acetate.

Another challenge is the reproducibility of biological replicates. Our own acetate-based preliminary shake flask and bioreactor experiments have shown that growth and productivity can differ significantly between replicates [115]. This is especially the case when defined media are used. Therefore, an even tighter process control needs to be applied than with glucose as a carbon source to improve reproducibility.

Additionally, process engineering can offer solutions for the issue of potentially toxic products. For instance, gas stripping has already been used to remove isopropanol from glucose-based fed-batch cultivations [143].

In situ extraction could be a means to obtain more hydrophobic chemicals as has been demonstrated with the sesquiterpene alcohol α-bisabolol [173]. Such an approach could also be used for β-caryophyllene produced from acetate.

The final challenge for process engineering is the coupling of acetate-based production to other processes. Syngas fermentation as a possible source for acetate was successfully tested with isopropanol [114]. Other processes could be coupled too, enabling continuous production chains from sustainable acetate sources to value-added chemicals. Coupled process steps like gas stripping may also alleviate product toxicity and increase product titers as shown for acetate-based production of acetone and the glucose-based production of isopropanol [113,143].

## 6. Outlook

Several topics still need to be addressed when it comes to acetate-based production.

The first issue concerns media additives. As of yet, yeast or beef extract in different concentrations is utilized for isopropanol, acetone, itaconic acid, or β-caryophyllene to name a few examples [33,60,113,114]. Even though these complex media additives may sometimes be a cheap nitrogen source, they can severely alter intracellular metabolite abundance. Intracellular amino acid pools were shown to be bigger in cells grown on complex media, while fatty acids, sugars, and sugar alcohols were more likely to be found in cells grown on defined media [174]. Changes in these metabolite pools can have varying effects on products, depending on their branching point from central carbon metabolism. Moreover, metabolic engineering also plays an important role to improve acetate-based production pathways. In this context, complex media additives might impede efforts to elucidate the effects of different metabolic engineering approaches on productivity.

Investigations concerning metabolic engineering approaches constitute another field of potential future work. Obtaining knowledge on factors influencing the productivity of chemicals derived from different metabolic intermediates is certainly paramount [152,153]. For instance, the identification of gene deletions that benefit the production of acetyl-CoA-derived chemicals would help develop a toolbox to improve existing acetate-based production. Moreover, establishing such a toolbox could further incentivize the upgrading of acetate into value-added chemicals that have not yet been produced in *E. coli*.

Additionally, the use of metabolomics combined with precise metabolic models on acetate might identify future targets for metabolic engineering. Models for the acetate overflow metabolism in *E. coli*, its reaction to oxygen, or the transition from glucose to acetate as growth substrate have already been established [175,176,177]. However, robust simulations for the growth and production on acetate are still lacking, but such simulations would provide a valuable tool for future metabolic engineering. This is especially the case if cofactor engineering becomes more tied into such models to alleviate energetic challenges that came along with using acetate as the sole carbon source [172].

To improve acid tolerance in general, further research and application of existing techniques could be a promising way. This includes studies on the expression of genes conferring acid tolerance, investigations concerning global regulators, or adaptive laboratory evolution experiments, which would also be applicable to enhance product tolerance [154,158,159,170].

Finally, developments that facilitate the industrial implementation of acetate-based production will be necessary. The establishment of continuous processes would be essential to make acetate industrially attractive. The combination of continuous acetate-based production with in situ product recovery would allow for high productivity while also maintaining high product titers. Such measures will be necessary to compete with petrochemical production, which already often employs continuous processes [178]. However, the challenges of toxic products and acetate tolerance still need to be overcome [143,168,179]. If the generation of acetate from sustainable sources is combined with its upgrading to value-added chemicals, quality variations of substrates like wastes must also be taken into account and might require clever process design to solve [180].

## 7. Conclusions

Microbial conversion of acetate into value-added products can pose an ecologically sustainable alternative to fossil fuels. To be economically competitive, further research on the metabolism and metabolic engineering of potential host organisms like *E. coli* is still required. In combination with efficient process design, this could lead the way towards “green” production of industrially relevant platform chemicals.

## Figures and Tables

**Table 1:** Reported specific growth rates on acetate for strains of *E. coli*, *V. natriegens*, and *P. aeruginosa.*

Organism & Strain	Acetate Concentration [mM]	Specific Growth Rate	Reference
*E. coli* MC4100	42	0.33 ± 0.05 h^−1^	[38]
*E. coli* BW25113	60	0.28 ± 0.03 h^−1^	[52]
*E. coli* W	169	0.46 h^−1^	[60]
*E. coli* W	85	0.37 ± 0.01 h^−1^	[61]
*E. coli* C	85	0.41 ± 0.01 h^−1^	[61]
*V. natriegens* DSM 759	42	0.45 ± 0.03 h^−1^	[48]
*P. aeruginosa* PAO1	20	0.80 ± 0.01 h^−1^	[49,50]

**Table 2:** List of enzymes involved in the central acetate metabolism of *E. coli*, *V. natriegens*, and *P. aeruginosa*; respective genes of enzymes or their subunits are given in brackets.

*E. coli*, *V. natriegens*, and *P. aeruginosa*		
Enzyme Number	Enzyme	Part in Metabolism
1	Acetate kinase (*ackA*)	Acetate uptake
2	Phosphotransacetylase (*pta*)	
3	Acetyl-CoA synthetase (*acs*)	
4	Citrate synthase (*gltA*)	TCA cycle
5	Aconitate hydratase (*acnA/acnB*)	
6	Isocitrate dehydrogenase (*icd*)	
7	α-ketoglutarate dehydrogenase (*sucA/sucB*)	
8	Succinyl-CoA synthetase (*sucC/sucD*)	
9	Succinate dehydrogenase (aerobic: *sdhCDAB*)/Fumarate reductase (anaerobic: *frdABCD)*	
10	Fumarate hydratase (aerobic: *fumA*/ anaerobic: *fumB*)	
11	Malate dehydrogenase (*mdh/mqo*)	
12	Isocitrate lyase (*aceA*)	Glyoxylate cycle
13	Malate synthase (*glcB*)	
14	Malate dehydrogenase (*maeA/maeB*)	Pyruvate metabolism
15	Phosphoenolpyruvate carboxykinase (ATP-dependent) (*pckA*)	
16	Pyruvate kinase (*pykA/pykF*)	
17	Pyruvate dehydrogenase (*aceE/aceF*)	
18	Pyruvate dehydrogenase (*poxB*)	
19	Pyruvate carboxylase (*pycA/pycB*)	*P. aeruginosa* specific; pyruvate metabolism

**Table 3:** List of enzymes involved in the central acetate metabolism of *Y. lipolytica*. Organelles are indicated in brackets (m = mitochondrion, c = cytoplasm, p = peroxisome).

*Y. lipolytica*		
Enzyme Number	Enzyme	Part in Metabolism
1	Acetyl-CoA synthetase (c)	Acetate uptake
2	Citrate synthase (m)	TCA cycle
3	Aconitate hydratase (m)	
4	Isocitrate dehydrogenase (m)	
5	α-ketoglutarate dehydrogenase (m)	
6	Succinyl-CoA synthetase (m)	
7	Succinate dehydrogenase (m)	
8	Fumarate hydratase (m)	
9	Malate dehydrogenase (m)	
10	Malate dehydrogenase (c)	Pyruvate metabolism
11	Pyruvate carboxylase (c)	
12	Pyruvate kinase (c)	
13	Pyruvate dehydrogenase (m)	
14	Aconitate hydratase (c)	Glyoxylate cycle
15	Isocitrate lyase (c)	
16	Malate synthase (p)	
17	Malate dehydrogenase (p)	
18	Malate dehydrogenase (c)	

**Table 4:** List of enzymes involved in the central acetate metabolism of *C. kluyveri.*

*C. kluyveri*	
Enzyme Number	Enzyme
1	Acetate kinase
2	Phosphotransacetylase
3	Alcohol dehydrogenase
4	Aldehyde dehydrogenase
5	Butyryl-CoA: Acetate CoA transferase
6	Acetoacetyl-CoA thiolase
7	3-Hydroxybutyryl-CoA dehydrogenase
8	3-Hydroxybutyryl-CoA dehydratase
9	Ferredoxin-dependent hydrogenase
Nfn	NADH-dependent reduced ferredoxin:NADP oxidoreductase
Rnf	Ferredoxin-NAD reductase complex

**Table 5:** List of enzymes involved in the acetate specific metabolic adaptions of sulfate-reducing bacteria.

Sulfate Reducers		
Enzyme Number	Enzyme	Pathway
1	ATP citrate lyase	Modified TCA cycle
2	Aconitate hydratase	
3	Isocitrate dehydrogenase	
4	2-Oxoglutarate synthase	
5	Succinyl-CoA:acetate CoA-transferase	
6	Succinate dehydrogenase	
7	Fumarate hydratase	
8	Malate dehydrogenase	
9	Acetate kinase	Reverse Wood–Ljungdahl pathway
10	Phosphotransacetylase	
11	Acetyl-CoA synthase	
12	Carbon monoxide dehydrogenase	
13	Methyltransferase	

**Table 6:** Examples of value-added compounds produced from acetate in different microbial host organisms (except *E. coli*).

Product	Organism	Comment	Reference
Lipids	*Y. lipolytica*		[97]
	*Cryptococcus curvatus ATCC 20509*	Study also screened various other oleaginous yeasts for acetate-based production	[98]
	*Rhodobacter* sp. KKU-PS1		[99]
	*Aurantiochytrium limacinum* SR21		[100]
Polyhydroxyalkanoate/ polyhydroxybutyrate	*Dinoroseobacter* sp. JL1447		[101]
	*Pseudomonas putida* KT2440		[37]
	*Synechocystis* PCC 6803	Acetate and glucose	[102]
	*Aeromonas hydrophilia* 4AK4	Metabolically engineered	[103]
	*Y. lipolytica* Po1 g	Heterologous pathway for PHB production introduced	[104]
Histidine	*Brevibacterium flavum* FERM1564 *(Corynebacterium glutamicum)*	Glucose added; uracil auxotroph mutant strain	[105]
Caproate	*Clostridium kluyveri* DSM 555	Ethanol as additional carbon source	[36]
Malic acid	*Aspergillus oryzae* DSM1863	Sequential culture with *Clostridium ljungdahlii* for acetate production from syngas	[106]
H_2_	*Rhodobacter sphaeroides* ATCC 17023	Glutamate as N-source	[107]
Electricity	*Cupriavidus basilensis* 9750	As part of a microbial fuel cell	[108]
Methane/biogas	Various methanogens in communities with acetogens	Different wastes/ biowastes as substrates for acetate and H_2_ production	[109,110,111]
Isotope labeled L-glutamate	*Brevibacterium flavum* ATCC 14067 *(Corynebacterium glutamicum)*	^13^C-acetate for labelled L-glutamate	[112]

**Table 8:** Enzymes of the central carbon metabolism of *E. coli* for glucose, glycerol, and acetate.

Enzyme Number	Enzyme	Part in Metabolism
1	Acetate kinase (*ackA*)	Acetate uptake
2	Phosphotransacetylase (*pta*)	
3	Acetyl-CoA synthetase (*acs*)	
4	Citrate synthase (*gltA*)	TCA cycle
5	Aconitate hydratase (*acnA/acnB*)	
6	Isocitrate dehydrogenase (*icd*)	
7	α-ketoglutarate dehydrogenase (*sucA/sucB*)	
8	Succinyl-CoA synthetase (*sucC/sucD*)	
9	Succinate dehydrogenase (*sdhCDAB*)	
10	Fumarate hydratase (aerobic: *fumA)*	
11	Malate dehydrogenase (*mdh/mqo*)	
12	Isocitrate lyase (*aceA*)	Glyoxylate cycle
13	Malate synthase (*glcB*)	
14	Malate dehydrogenase (*maeA/maeB*)	Pyruvate metabolism
15	Phosphoenolpyruvate carboxykinase (ATP-dependent) (*pckA*)	
16	Pyruvate kinase (*pykA/pykF*)	
17	Pyruvate dehydrogenase (*aceE/aceF*)	
18	Pyruvate dehydrogenase (*poxB*)	
19	Glucokinase (*glk*)or PTS system	Glycolysis; glucose
20	Glucose-6-phosphate isomerase (*pgi*)	
21	ATP-dependent 6-phosphofructokinase (*pfkB*)	
22	Fructose-bisphosphate aldolase (*fbaB*)	
23	Triosephosphate isomerase (*tpiA*)	Glycolysis
24	Glyceraldehyde-3-phosphate dehydrogenase A (*gapA*)	
25	Phosphoglycerate kinase (*pgk*)	
26	2,3-Bisphosphoglycerate-dependent/independent phosphoglycerate mutase (*gpmA/gpmI*)	
27	Enolase (*eno*)	
28	Glycerol kinase (*glpK*)	Glycolysis; glycerol
29	Aerobic glycerol-3-phosphate dehydrogenase (*glpD*)	

## Supplementary Materials

Supplementary materials can be found at https://www.mdpi.com/1422-0067/21/22/8777/s1.

## Abbreviations

TCA cycle	Tricarboxylic acid cycle
MTY	Maximum theoretical yield
MTCY	Maximum theoretical carbon yield
